# Native T1-mapping using cardiovascular magnetic resonance detects myocardium at risk during the first week following myocardial infarction in a swine model and in patients - comparison to contrast-enhanced cine steady-state free precession

**DOI:** 10.1186/s12872-026-05507-3

**Published:** 2026-01-22

**Authors:** Theodor Lav, David Nordlund, Christos Xanthis, Jonathan Berg, Sebastian Bidhult, Anthony H. Aletras, Robert Jablonowski

**Affiliations:** 1 Clinical Physiology, Department of Clinical Sciences Lund, Lund University, Skane University Hospital, Lund, 221 85 Sweden; 2 Laboratory of Computing, Medical Informatics and Biomedical-Imaging Technologies, School of Medicine, Aristotle University of Thessaloniki, Thessaloniki, Greece

**Keywords:** Native T1-mapping, Contrast-enhanced SSFP, Myocardium at risk, Area at risk, Infarct size, Late gadolinium enhancement, ST-elevation myocardial infarction, Pig model

## Abstract

**Background:**

Myocardium at risk (MaR) can be evaluated by cardiovascular magnetic resonance (CMR) imaging using contrast-enhanced steady state free precession (CE-SSFP) in patients after ST-elevation myocardial infarction (STEMI). However, CE-SSFP utilizes gadolinium contrast, which is contraindicated in patients with severe renal insufficiency. Native T1-mapping is a non-contrast CMR method which has been shown feasible in assessing MaR, enabling patients with gadolinium contrast contraindications to be examined. However, native T1-mapping data have been presented in the sub-acute phase suggesting to also depict infarct size (IS), as assessed by late gadolinium enhancement (LGE). Therefore, it is unclear whether native T1-mapping depicts MaR or IS during the first week after reperfusion. We hypothesized that native T1-mapping agrees with MaR as assessed by CE-SSFP and overestimates IS as assessed by LGE in an experimental pig model and in patients during the first week after STEMI.

**Methods:**

A retrospective analysis was performed using CMR images from an infarct/reperfusion experimental pig model. CMR imaging was performed at 2 h, 24 h and 7 days after reperfusion in a serially imaged group (*n* = 7) and at 4 days in a single-timepoint imaged group (*n* = 4). Also, STEMI patients with a single vessel LAD occlusion (*n* = 11) were CMR imaged between 3 to 7 days after reperfusion. Native T1-mapping MOLLI, CE-SSFP and LGE were acquired for each scan in both animals and patients. In animals, images with an additional T1-mapping sequence, SASHA, were acquired. Enhanced areas on T1-maps, CE-SSFP and LGE images were quantified and compared.

**Results:**

*In pigs*, native T1-mapping MOLLI agreed with CE-SSFP in the single-timepoint- and serially imaged groups (bias: 0.3 ± 6.6% (mean ± 2SD), and 0.9 ± 18%), respectively. Native T1-mapping SASHA also agreed with CE-SSFP in the serially imaged group (bias: -0.1 ± 18%). However, MOLLI overestimated IS by LGE in pigs in the serially- and single-timepoint imaged groups (bias: 21 ± 26%, and 18 ± 17%), respectively. Similar results were seen in patients (MOLLI vs. CE-SSFP: 0.8 ± 7.5%, and MOLLI vs. LGE: 31 ± 22%).

**Conclusion:**

Our findings suggest that native T1-mapping agrees with CE-SSFP during the first week after myocardial infarction when evaluating MaR. Also, native T1-mapping overestimates the LGE hyperintense area, indicating that native T1-mapping does not primarily depict infarct size.

**Supplementary Information:**

The online version contains supplementary material available at 10.1186/s12872-026-05507-3.

## Introduction

Cardiovascular magnetic resonance (CMR) imaging is used for evaluating myocardium at risk (MaR) and infarct size (IS) after ST-segment elevation myocardial infarction (STEMI) [1]. The evaluation of myocardial infarction with CMR is used in cardioprotective interventional research aiming to decrease reperfusion injury after percutaneous coronary intervention (PCI) [1–3]. The effect of a cardioprotective intervention can be measured by myocardial salvage index (MSI) for which both IS and MaR needs to be known [1]. Infarct size is evaluated by late gadolinium enhancement (LGE) which requires the administration of gadolinium (Gd) contrast agent [4].

Different CMR sequences can be used to depict MaR, including contrast-enhanced steady-state free precession (CE-SSFP), T2-weighted (T2W) imaging and T2-mapping [5–8]. CE-SSFP has been compared to T2W imaging in STEMI patients with better diagnostic performance of MaR than T2W imaging [9]. In addition, CE-SSFP enables assessment of both volumetrics and extent of edema using the same images [5]. However, the technique requires administration of Gd contrast agent which is associated with the risk of developing nephrogenic systemic fibrosis or nephrotoxicity in patients suffering from severe renal insufficiency and is contraindicated during pregnancy [10]. A non-contrast protocol could also reduce scan time and enable more post-STEMI patients to be examined by CMR.

A non-contrast method for evaluating infarct characteristics is native T1-mapping, which is based on T1 values from a combination of myocytes and myocardial extracellular volume [11, 12]. This method has been shown to agree with the edematous extent measured by T2-mapping in patients during the first week at 2–4 days after infarction [13]. However, data demonstrating native T1-mapping to correlate with IS in the sub-acute phase after STEMI have also been presented [14]. Hence, it is still unclear if native T1-mapping depicts MaR or IS during the first week after reperfusion. Furthermore, native T1-mapping has not been validated against CE-SSFP.

Therefore, we aimed to investigate whether native T1-mapping agrees with MaR assessed by CE-SSFP or IS by LGE during the first week after STEMI. We hypothesized that native T1-mapping agrees with MaR assessed by CE-SSFP and overestimates IS as assessed by LGE in an experimental pig model and in patients during the first week after STEMI.

## Materials and methods

### Experimental protocol

**Fig. 1:**
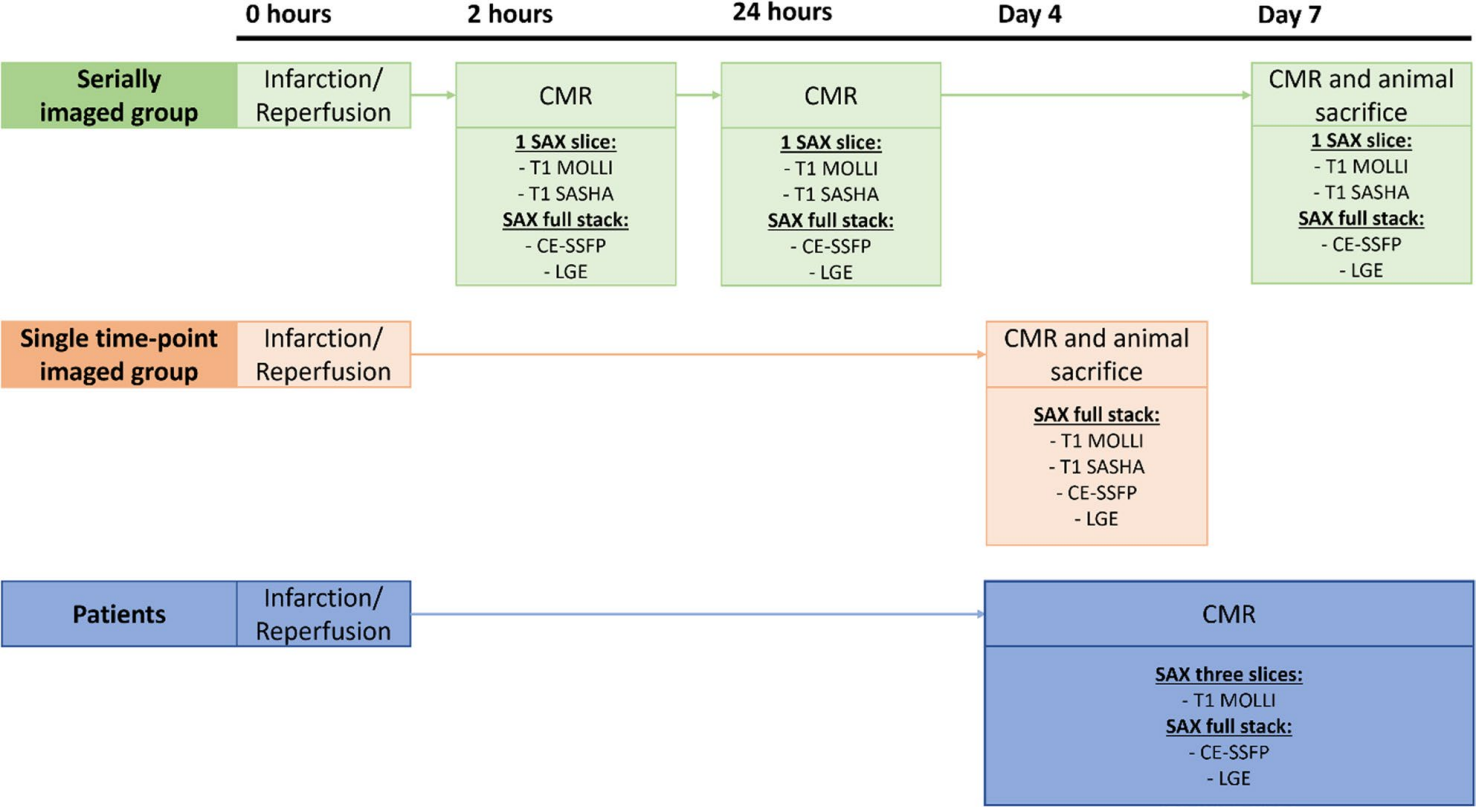
Study design. Pigs were separated into the serially- and single timepoint imaged groups. The serially imaged group (*n* = 7) was CMR imaged at 2 h, 24 h and 7 days after infarct/reperfusion. The single-timepoint imaged group (*n* = 4) was CMR imaged at 4 days after infarct/reperfusion. All animals were sacrificed after their final CMR imaging timepoint. Patients were CMR imaged 3–7 days after myocardial infarction. CE-SSFP = Contrast enhanced steady-state free precession. CMR = cardiovascular magnetic resonance. LGE = Late gadolinium enhancement. MOLLI = Modified Look-Locker Inversion recovery. SASHA = Saturation recovery single-Shot Acquisition. SAX = Short-axis

All animal experiments were conducted between 2018 and 2019 and approved by the Swedish Board of Agriculture through the Malmö/Lund ethics committee on Animal Testing (registration number 5681/2017). Inclusion criteria were pigs subjected to a left anterior descending artery (LAD) infarct. Eleven pigs were included from two previous experiments with an infarct/reperfusion model. The first group (*n* = 7) consisted of the control group in a study investigating the hypothermic effect on infarct development [15]. Pigs were serially imaged after infarct induction in this group. Three control animals developed non-treatable arrythmia or circulatory collapse during anesthesia and were therefore not included in the analysis of the serially imaged group. The second group (*n* = 4) consisted of all animals in an experiment investigating MaR during the first week after infarction/reperfusion. Animals in this group were imaged at a single timepoint four days after infarction/reperfusion where dynamics of the edematous MaR are stable. No adverse events occurred in this group. To comply with ARRIVE guidelines, an experimental protocol was prepared before the study was conducted. The protocol was not formally registered. Also, the methods of infarct induction and reperfusion were the same for both groups. Acclimatization in groups of at least two pigs was ensured for at least one day before the experimental procedure. Sedation, anesthesia, surgical preparation, and invasive procedures were performed in the animal laboratory. Sedation was performed with an intramuscular injection, followed by intubation, general anesthesia and monitoring of the animal (see Supplementary material, Table S1 for sedation and anesthesia protocol). Femoral arterial and venous access was established using Seldinger technique. The animal was monitored by ECG, intraarterial blood pressure, intravenous temperature, EtCO_2_ and saturation throughout the experiment. A balloon was inserted via the arterial femoral access and inflated in the left anterior descending artery (LAD) distal to the first diagonal branch for 40 min to provide an infarct approximately 50% of the MaR [16]. Fentanyl was administered prior to and during balloon inflation to counteract any ischemic pain. Angiograms were used to ensure the cessation and return of blood flow during the procedure. Following infarct/reperfusion, in-vivo CMR was performed at 2 h, 24 h, and 7 days in the serially imaged group and at 4 days in the single-timepoint imaged group, see Fig. 1 [15]. Each animal was awakened and brought together with other pigs after the scan at 2 h and 24 h in the serially imaged group. For each in-vivo CMR timepoint Gd-DOTA (Dotarem, Guerbet, Roissy, France) was administered intravenously. After the final CMR-imaging, the animals were terminated when unconscious under anesthesia by inhalation of Isoflurane. The euthanasia was performed injecting an overdose of pentobarbital-based anaesthetic (250 mg/kg, Euthasol, Virbac, Kolding, Denmark) intravenously. Death of the animal was ensured by cessation of electrical discharge on the ECG. Details of husbandry and humane endpoints are stated in Supplementary material, Table S2 and S3, respectively. All animals were privately owned by a local farm with an agreement of delivery of animals for experimental usage by the university. Informed consent for conducting experiments by the owner was obtained as part of the agreement with the farm.

### In-vivo experimental CMR

CMR was performed for all animals on a 1.5 T MRI scanner (MAGNETOM Aera, Siemens Healthineers, Forchheim, Germany). CMR images with obvious image artifacts were excluded. We performed pre-contrast cine imaging, consisting of a short-axis stack and 2-, 3- and 4 chamber images, that were used as an anatomic reference to select native T1 mapping slices.

*T1-mapping.* A research pulse sequence was used to perform pre-contrast native T1-mapping using a Modified Look-Locker Inversion recovery (MOLLI) sequence with the following parameters: MOLLI 5s(3s)3s, flip angle 35º, repetition time (TR) 2.6 ms, echo time (TE) 1.1 ms, inversion times (TI) varied depending on heart rate, and voxel size 1.5 × 1.5 × 8 mm^3^ [17]. MOLLI used two inversion pulses with a starting TI of 104 ms and a TI increment of 80 ms. In addition, a research sequence was used to perform Saturation recovery single-Shot Acquisition (SASHA) native T1-mapping with the following typical parameters: flip angle 70º, TR 2.6 ms, TE 1.1 ms, saturation times (TS) 110–320 ms, 110–415 ms, 110–550 ms or 110–700 ms depending on heart rate with increment depending on the maximum TS, and voxel size 1.5 × 1.5 × 8 mm^3^. One SAX slice of the LV was acquired with native T1-mapping for each animal and timepoint in the serially imaged group.

*CE-SSFP.* A full short-axis stack of CE-SSFP images was acquired six minutes after Gd contrast administration with an acquisition time of ~ 6 min [18]. CE-SSFP cine end-expiratory breath-held segmented acquisition was used with the following parameters: GRAPPA acceleration factor 2, flip angle 60º, echo time (TE) 1.1 ms, repetition time (TR) 2.6 ms, temporal resolution 36.5 ms, bandwidth 1184 Hz/pixel, 192 pixels along the frequency-encoding direction, and voxel size 1.7 × 1.7 × 8 mm^3^.

*LGE.* A full stack of late gadolinium enhancement short- and long-axis images were acquired 15–20 min following gadolinium contrast administration using a multi-slice, non-cine single-shot motion corrected phase-sensitive inversion recovery sequence (PSIR). The following sequence parameters were used: flip angle 50º, TR 2.8 ms, TE 1.2 ms, 8 averages in the multi-shot acquisition, GRAPPA acceleration factor 2, readout duration 203.8 ms, bandwidth 977 Hz/pixel with 192 pixels along the frequency-encoding direction, no slice gap and voxel size 1.4 × 1.9 × 8 mm^3^. Late gadolinium enhancement short- and long-axis images were acquired using a multi-slice motion corrected phase-sensitive inversion recovery sequence (PSIR) after 15–20 min following CE-SSFP sequence. The following typical parameters were used: flip angle 50º, TR 2.8 ms, TE 1.2 ms, no slice gap and voxel size 1.4 × 1.9 × 8 mm^3^ [4, 19]. Scouts were used to determine appropriate TI for nulling of the myocardium.

### Patient CMR protocol

The patient data collection was approved by the Malmö/Lund Ethical Review Board (application number 2013/900). Informed written consent was received from all patients before inclusion. Inclusion criteria consisted of patients with STEMI and a confirmed single vessel LAD occlusion treated with primary PCI. Patient data were collected from eleven patients with STEMI and a confirmed LAD occlusion treated with primary PCI. Imaging by CMR was performed at 3–7 days after reperfusion. The same native T1-mapping MOLLI (5s(3s)3s) research sequence as in the animals was performed in three SAX slices of the LV before contrast administration with the following parameters: flip angle 35º, repetition time (TR) 2.4 ms, echo time (TE) 1.0 ms, and voxel size 1.9 × 2.4 × 8 mm^3^ [17]. Six minutes after Gd contrast administration, a full short-axis stack of CE-SSFP images was acquired with an acquisition time of ~ 6 min and the following parameters: flip angle approximately 50º, repetition time (TR) 2.6 ms, echo time (TE) 1.1 ms, and voxel size 2.0 × 2.0 × 8 mm^3^. The same LGE PSIR sequence parameters were used as for the in-vivo sequence protocol mentioned above. In the analysis between native T1 and CE-SSFP, a total of two patients were excluded due to motion artifacts on native T1 and CE-SSFP images, yielding a total of 9 patients for the final analysis. In the analysis between native T1 and LGE, an additional patient was excluded due to no LGE on the investigated slice that corresponded to the images position of the native T1 image, thus yielding a total of 8 patients for the final analysis. Each T1 mapping stack contained 3 SAX-slices except for one stack with only one SAX slice due to clinical examination time constraints.

### Image analysis

Analysis of all CMR images was performed in the software Segment version 3.3 R9405 (http://segment.heiberg.se) [20]. The LV endocardium and epicardium were quantified manually excluding the papillary muscles in all SAX images. The corresponding CE-SSFP- and LGE-image slices were matched with the native T1-maps MOLLI and SASHA SAX image slices in the serially imaged group and in patients. In the single-timepoint imaged group full SAX stacks of CE-SSFP, native T1 maps with MOLLI and SASHA and LGE were acquired and therefore no slice matching was necessary. Four animals with a full stack SAX slices of CE-SSFP, LGE and native T1-mapping MOLLI and SASHA were included. In one animal of the single-timepoint imaged group no SASHA SAX stack was acquired. Quantification of the enhanced areas in the native T1 mapping images was performed manually. Notably, there is no current reference standard for quantification of edema in T1-maps today. This may impact the assessment of the size of the enhanced areas on the T1-map [21]. Myocardium at risk was defined in the CE-SSFP images by delineating and averaging enhanced areas at end-systole and end-diastole according to a previous validated method [5]. The infarct size was quantified by using the Expectation Maximization, weighted intensity, a priori information (EWA) algorithm with manual adjustments [22]. Areas with low signal within the enhanced extent of edema on the T1-maps, CE-SSFP and infarct areas on LGE-images represent microvascular obstruction and were included in the quantified areas. Quantified enhanced extents were reported as percent of total slice area in the serially imaged group, and percent of LV in the single-timepoint imaged group and in patients. All quantifications were performed blinded by one cardiologist with 12 years of CMR-imaging experience and in a subset by a second observer with 10 years of CMR experience. CE-SSFP-, native T1 mapping-, and LGE-images were segmented separately and randomly to reduce possible bias from segmenting images of the same case consecutively. Exclusion criteria constituted of (a) image slices with severe artefacts interfering with the LV myocardial wall and (b) SAX slices negative for infarction or edema in the LGE or CE-SSFP images for pigs or patients.

### Statistical analysis

Results were acquired through analysis with R V4.2.2 [23]. Each individual animal or patient was considered one experimental unit. CE-SSFP and LGE were defined as reference methods for MaR and IS, respectively. Native T1-maps by MOLLI and SASHA were compared to each reference method with a mixed model linear regression adjusted for repeated measures and Spearman correlation. Modified Bland-Altman analyses were performed to assess the agreement and interchangeability between native T1-maps and MaR or IS [24], with correction for repeated measures in the serially imaged group yielding wider limits of agreement. In the modified Bland-Altman analyses CE-SSFP and LGE were used as reference standard for MaR and IS respectively depicted on the x-axis [25]. Bias and limits of agreement were expressed as mean ± 2SD. Differences with a p-value < 0.05 indicated statistical significance. Since the study is retrospective and investigating agreement between methods, no sample size calculation or randomization was performed prior to or during the study. The manuscript was written according to ARRIVE guidelines [26].

## Results

### Experimental model

Characteristics of the pig model are demonstrated in Supplementary table S4. In the serially imaged group, 6 out of 7 animals with one LV SAX slice of CE-SSFP, LGE and native T1-mapping MOLLI and SASHA images were included for each timepoint (2 h, 24 h and 7 days) adding up to *n* = 18 slices in total. One animal was excluded due to no infarct or MaR in the investigated SAX slices. In the single-timepoint imaged group, a total of *n* = 4 and *n* = 3 LV SAX slices or stacks were compared between MOLLI or SASHA against CE-SSFP, respectively.

**Fig. 2:**
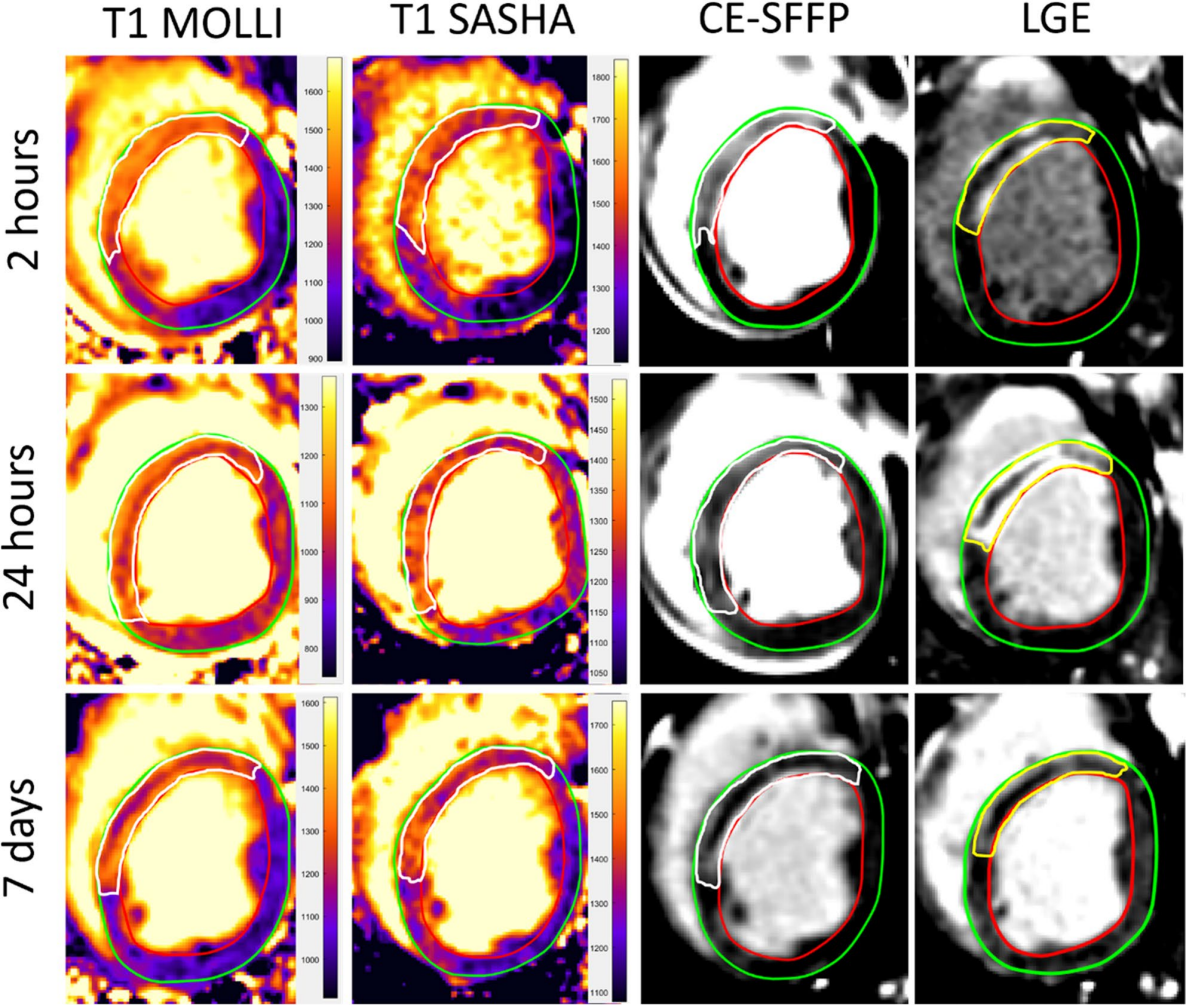
Example images from a pig. Quantification of hyperenhanced myocardial areas in short-axis slices of the left ventricle in the same animal from the serially imaged group at 2 h, 24 h and 7 days. The white demarcations are defined as myocardium at risk (MaR) in Contrast-enhanced steady state free precession (CE-SSFP) cardiovascular magnetic resonance (CMR) images and native T1-maps by MOdified Look-Locker Inversion recovery (MOLLI) and SAturation recovery Single-Shot Acquisition (SASHA) CMR sequences. The yellow demarcation in the short axis slices with late gadolinium enhancement (LGE) depicts infarct size. CE-SSFP = Contrast enhanced steady-state free precession. CMR = cardiovascular magnetic resonance. LGE = Late gadolinium enhancement. MOLLI = Modified Look-locker Inversion recovery. SASHA = Saturation recovery single-Shot Acquisition

**Fig. 3:**
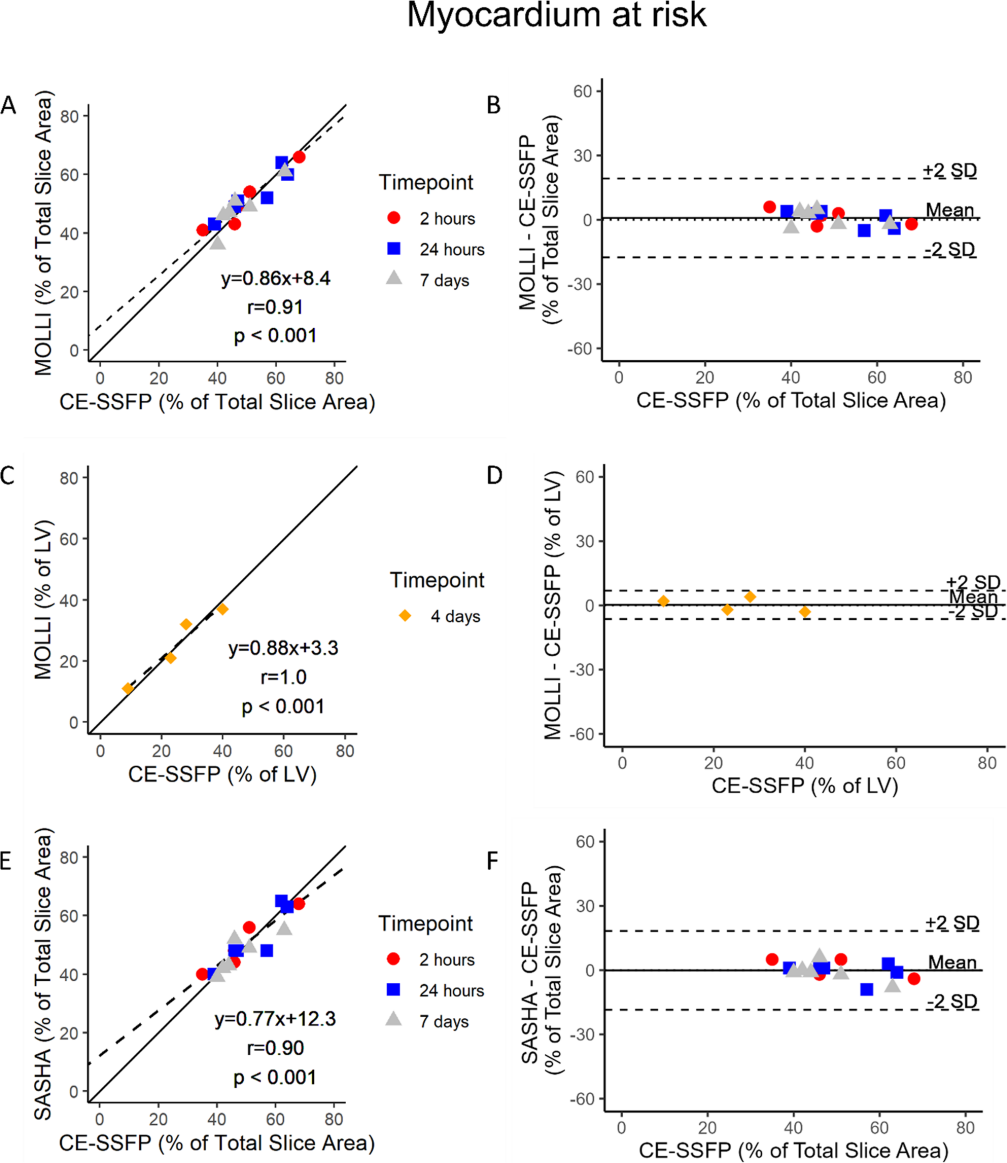
T1-mapping vs. CE-SSFP in pigs. Regression- and modified Bland-Altman analyses comparing MOdified Look-Locker Inversion recovery (MOLLI) against Contrast-enhanced steady state free precession (CE-SSFP) as reference by single-slice analyses at 2 h, 24 h and 7 days (**A** and **B**). Full stack short-axis analyses at 4 days after reperfusion for MOLLI against CE-SSFP in the single-timepoint imaged group are also presented (**C** and **D**). The same comparisons are presented for native T1-mapping Single-Shot Acquisition (SASHA) in the serially imaged group (**E** and **F**). The solid and broken lines depict the line of identity and the regression lines respectively. CE-SSFP = Contrast enhanced steady-state free precession. CMR = cardiovascular magnetic resonance. LV = Left ventricle. MOLLI = Modified Look-locker Inversion recovery. SASHA = Saturation recovery single-Shot Acquisition

*Myocardium at Risk.* Examples of native T1-mapping and CE-SSFP images are shown in Fig. 2. Correlation and agreement between native T1-mapping MOLLI and SASHA against CE-SSFP as reference method for quantification of MaR is shown in Fig. 3. Spearman correlation coefficients in the serially imaged group with single-slice based comparisons for native T1-mapping sequences MOLLI and SASHA against CE-SSFP were *r* = 0.91 and *r* = 0.90, respectively, with *p* < 0.001 for both. Bias and agreement between native T1-mapping and CE-SSFP was calculated by subtraction between the enhanced extent estimates and demonstrated 0.9 ± 18% for MOLLI, and − 0.1 ± 18% for SASHA. Full-stack comparisons for the single-timepoint imaged group of MOLLI against CE-SSFP as reference showed a correlation and bias of *r* = 1.0 (*p* < 0.001) and 0.3 ± 6.6 respectively. Interobserver agreement in a subset of two pigs at 2 h and 7 days for MOLLI, SASHA and CE-SSFP were 1.3 ± 9.7%, −4.2 ± 12% and − 3.2 ± 14%, respectively. Results from native T1-mapping SASHA against CE-SSFP in the single-timepoint imaged group are presented in Supplementary Figure S1, panels A and B.

**Fig. 4:**
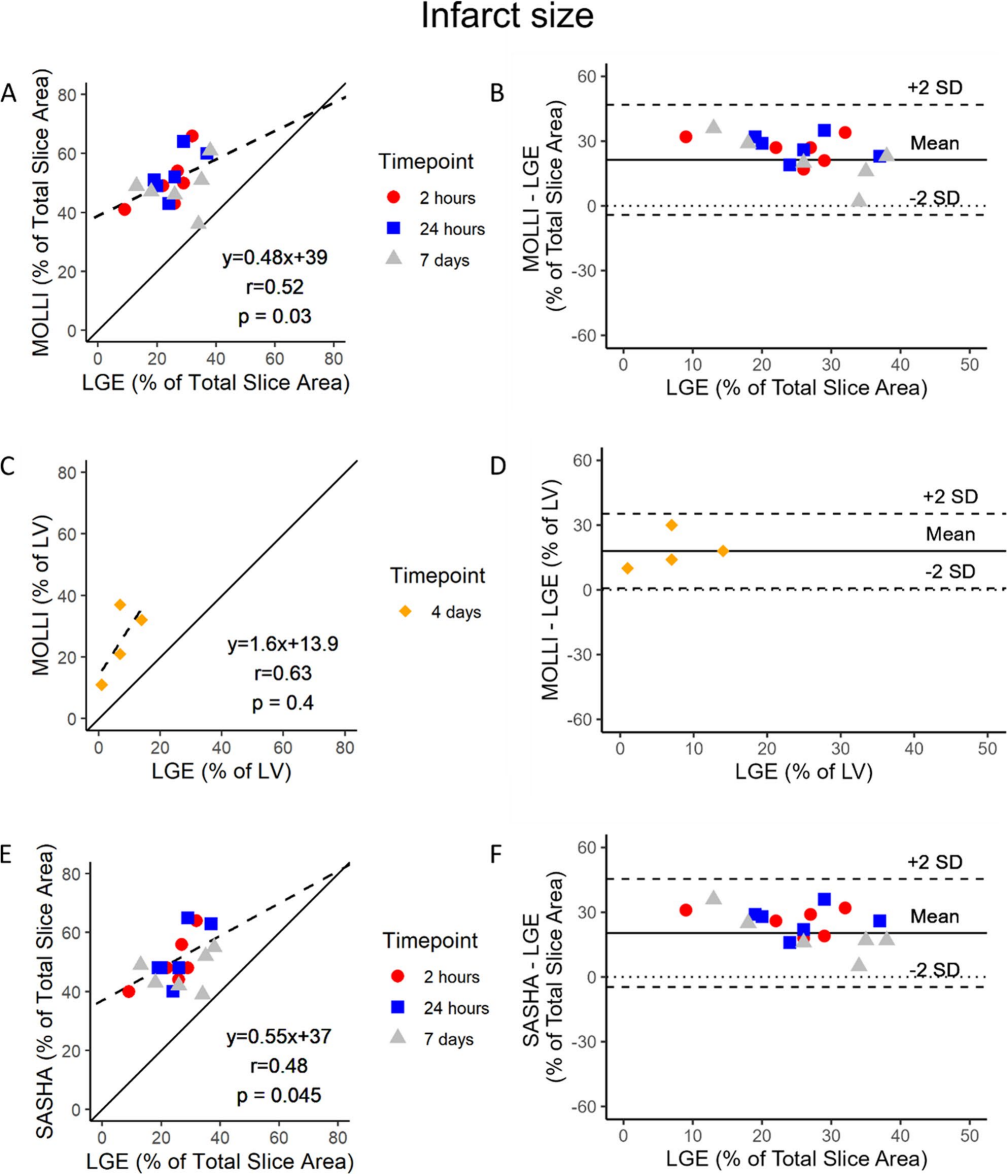
T1-mapping vs. LGE in pigs. Regression- and modified Bland-Altman analyses comparing MOdified Look-Locker Inversion recovery (MOLLI) against Late gadolinium enhancement (LGE) as reference by single-slice analyses at 2 h, 24 h and 7 days (**A** and **B**). Full stack short-axis analyses at 4 days after reperfusion for MOLLI against LGE in the single-timepoint imaged group are also presented (**C** and **D**). The same comparisons are presented for native T1-mapping Single-Shot Acquisition (SASHA) in the serially imaged group (**E** and **F**). The solid and broken lines depict the line of identity and the regression lines respectively. CMR = cardiovascular magnetic resonance. LGE = Late gadolinium enhancement. LV = Left ventricle. MOLLI = Modified Look-locker Inversion recovery. SASHA = Saturation recovery single-Shot Acquisition

*Infarct Size.* Examples of native T1-mapping and LGE images are shown in Fig. 2. Correlation and agreement between native T1-mapping and LGE as reference method for quantification of IS are shown in Fig. 4. Spearman correlation coefficients in the serially imaged group for MOLLI and SASHA against LGE were *r* = 0.52, *p* = 0.03, and *r* = 0.48, *p* = 0.045, respectively, and thus lower than the correlation between native T1-mapping against CE-SSFP. Bland-Altman bias and agreement between native T1-mapping and LGE were calculated by subtraction between the enhanced extent estimates and were 21 ± 26% for MOLLI, and 20 ± 25% for SASHA. Results from native T1-mapping SASHA against LGE in the single-timepoint imaged group are presented in Supplementary Figure S1, panels C and D.

### Patients

**Fig. 5:**
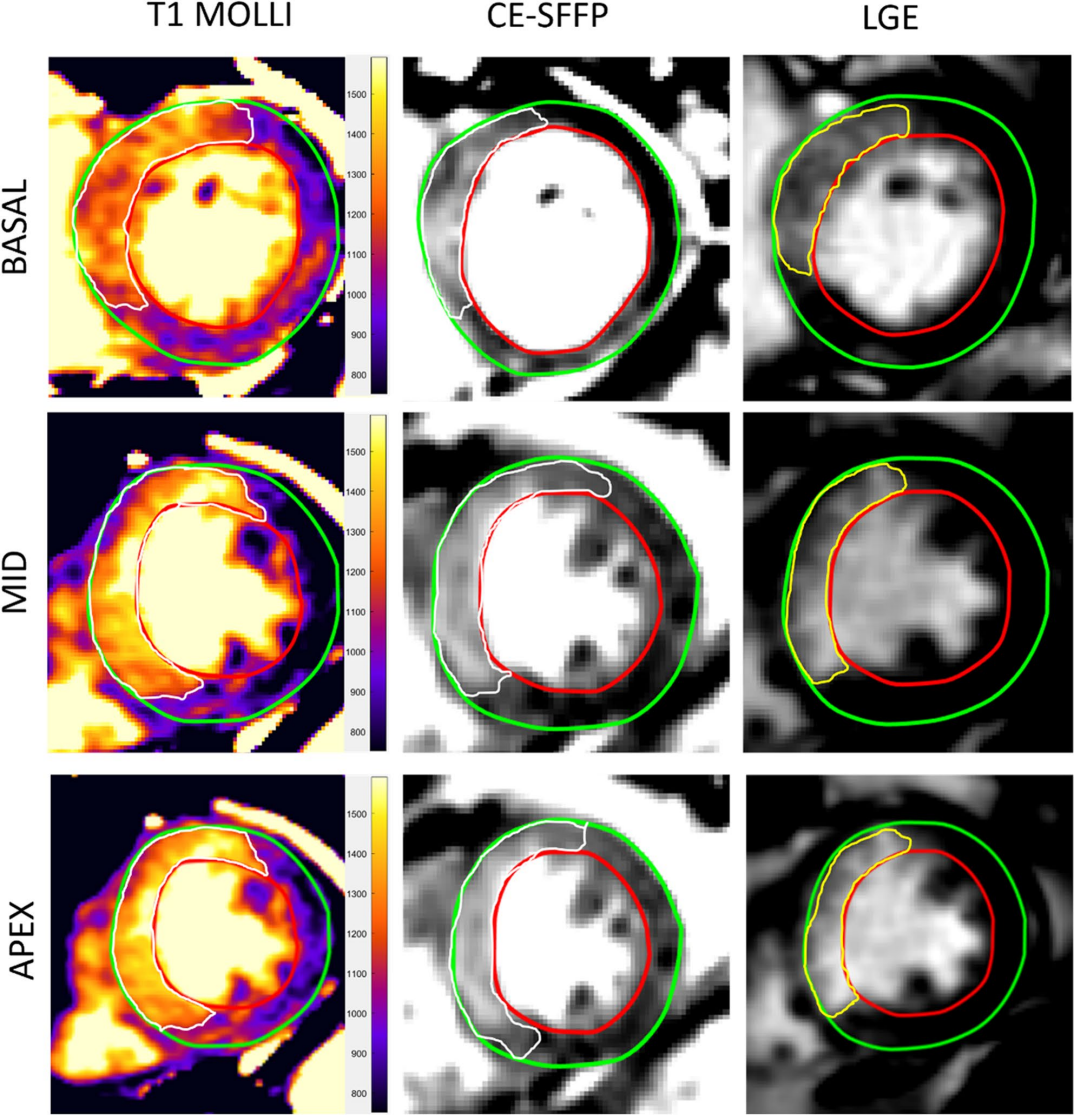
Example images from a patient. Quantification of hyperenhanced myocardial areas in 3 short-axis slices of the left ventricle in the same patient. The white quantifications were defined as myocardium at risk (MaR) in native T1-maps by MOdified Look-Locker Inversion recovery (MOLLI) cardiovascular magnetic resonance (CMR) images and Contrast-enhanced steady state free precession (CE-SSFP) CMR images. Infarct size was demarked by the yellow line in the late gadolinium enhancement (LGE) images. CE-SSFP = Contrast enhanced steady-state free precession. CMR = cardiovascular magnetic resonance. LGE = Late gadolinium enhancement. MOLLI = Modified Look-locker Inversion recovery

Characteristics of the patient study population are listed in Table 1. Examples of native T1-mapping, CE-SSFP and LGE images for patients are shown in Fig. 5.

**Table 1:** Patient characteristics. Data presented as absolute number ± standard deviation or percent in parenthesis

Characteristics	*n* (%)
Number of patients	11
Female	1 (9)
Age (years)	63 ± 10
BMI	27 ± 4
Smoking	2 (18)
Diabetes	1 (9)
Hypertension	5 (45)

**Fig. 6:**
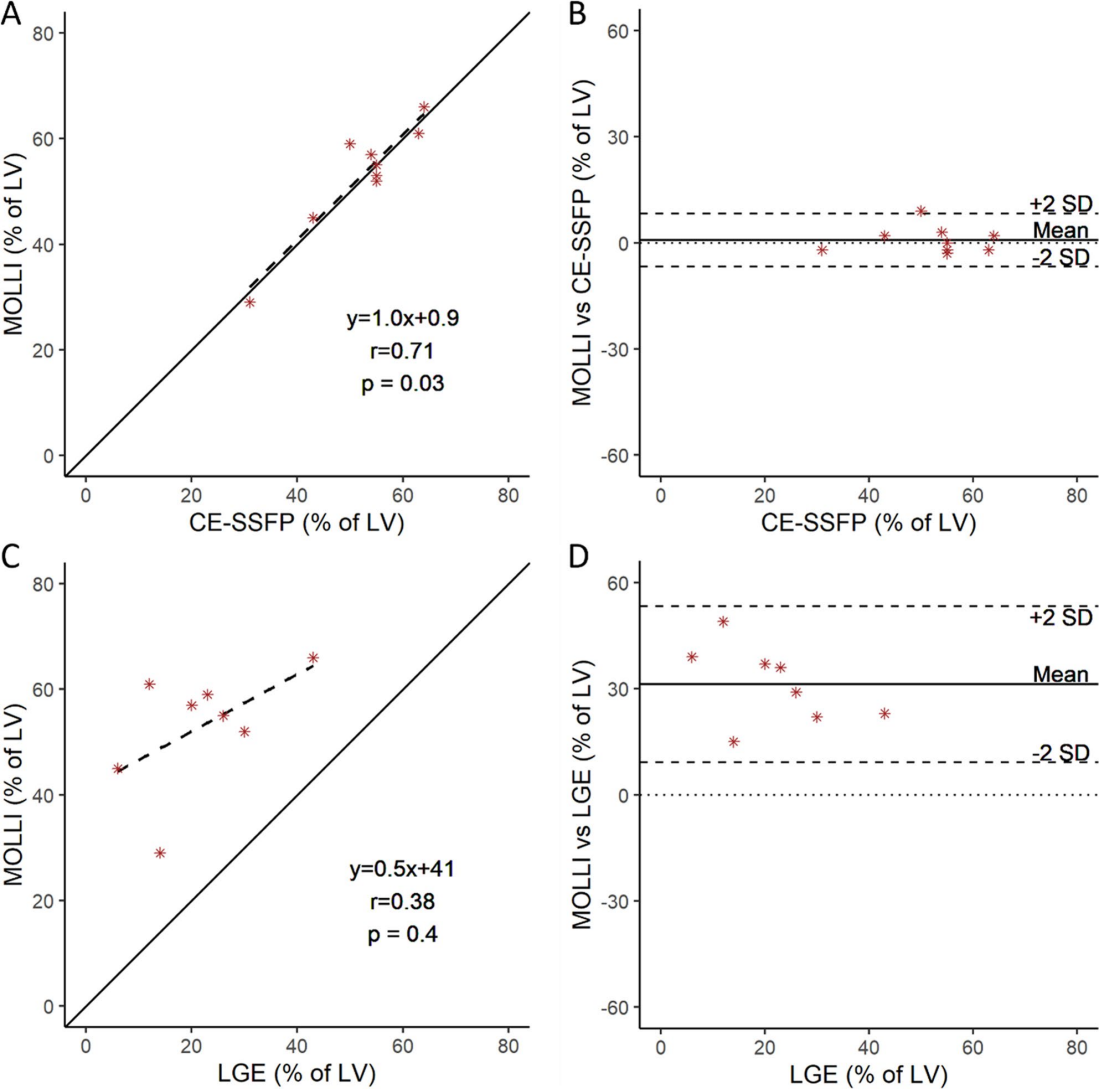
T1-mapping vs. CE-SSFP and LGE in patients. Linear regression (dotted line) with Spearman correlation between manual quantification of Contrast-enhanced steady state free precession (CE-SSFP) images, and manual quantification of native T1-mapping MOdified Look-Look Inversion recovery (MOLLI) images (**A**) in patients at 3–7 days after reperfusion. A modified Bland-Altman plot is shown with difference in native T1-mapping MOLLI versus CE-SSFP (**B**). Linear regression (dotted line) with Spearman correlation between quantification by the EWA algorithm of Late gadolinium enhancement (LGE) images, and manual quantification of native T1-mapping MOLLI images (**C**). A modified Bland-Altman plot with difference in native T1-mapping MOLLI versus LGE (**D**). The solid and broken lines depict the line of identity and the regression lines respectively. CE-SSFP = Contrast enhanced steady-state free precession. LGE = Late gadolinium enhancement. LV = Left ventricle. MOLLI = Modified Look-Locker Inversion recovery

*Myocardium at Risk.* A linear regression and Bland-Altman plot for native T1-mapping MOLLI versus CE-SSFP is shown in Fig. 6A and B. The correlation between native T1-mapping and CE-SSFP was *r* = 0.71, *p* = 0.03. Bias and agreement, calculated as previously mentioned, were 0.8 ± 7.5% of the LV SAX slice in the evaluation of MaR.

*Infarct size.* A linear regression and Bland-Altman plot for native T1-mapping MOLLI versus LGE is shown in Fig. 6C and D. The correlation between native T1-mapping and LGE was *r* = 0.38, *p* = 0.4. Bias and agreement, calculated as mentioned above, were 31 ± 22% of the LV SAX slice in the evaluation of IS. Hence, the hyperintense area in the T1-mapping sequence was overestimating the infarct size.

## Discussion

Our findings suggest that non-contrast native T1-mapping using MOLLI and SASHA agrees with CE-SSFP the first week after STEMI when evaluating MaR in vivo and in patients. Also, native T1-mapping is shown to overestimate the LGE hyperintense area, indicating the method to not primarily depict IS but rather both the infarcted area and the surrounding edematous area. The finding that native T1 mapping agrees with CE-SSFP and not LGE is aligned with previous research showing that T1 reflects increased water content as in myocardial edema rather than irreversible injury [1, 27]. The results from this study support the assessment of MaR by a non-contrast protocol in patients with contraindication to Gd contrast agent during the first week post-STEMI, enabling these patients to become available for inclusion in interventional cardioprotective studies [10].

### Native T1-mapping and myocardial edema

The detection of MaR is based on that ischemic myocardium becomes edematous after perfusion is restored [6, 28, 29]. Altered T1-values in myocardial tissue with non-reversible injury was initially shown to be related to tissue water content [30, 31]. This relationship was shown to be more evident for T2 than T1 [32]. After development of new more precise mapping sequences, however, native T1-mapping was validated against T2-mapping experimentally in dogs and in patients [17, 27, 33]. Contrast-enhanced SSFP offers an additional method to evaluate the extent of MaR during post-contrast cine imaging [5, 8]. Our experimental data compared native T1-mapping against CE-SSFP and showed similar bias to a prior validation study that compared native T1-mapping against T2-mapping and microspheres for detection of MaR in a canine model [27]. Therefore, our study further strengthens the evidence by demonstrating agreeing results despite adjustments for repeated measures, and full stack data. In addition, our study shows that the enhanced extent by native T1-mapping and CE-SSFP agree during multiple timepoints from 2 h to 7 days in vivo. This indicates native T1-mapping to be suitable for assessing the edematous extent during the first week post-infarction. This is in line with earlier studies demonstrating the edematous extent to depict MaR during the first week after reperfusion [9]. However, the serial pig group was used to test interchangeability between timepoints and not to quantify the temporal evolution of MaR or IS given slice-position variability. In our study we used a single timepoint imaged group with imaging at 4 days in accordance with the imaging timepoint of clinical post-STEMI patients, as the edema is stable during 3–7 days post-revascularization [1]. However, future studies are needed to see how T1-mapping compares to CE-SSFP and LGE at later time points after STEMI. As the myocardial edema is resorbed with time the remaining T1-elevation could be caused by scar tissue as seen during the subacute post-infarct phase [14]. It remains unknown at what time native T1-mapping starts to only depict IS. Our comparison between native T1-mapping and CE-SSFP in patients have similar bias and precision compared to earlier studies validating native T1-mapping against T2-mapping in patients at comparable post-reperfusion time [13, 33]. Inter-patient variability could affect the correlation between native T1 mapping and CE-SSFP as, given the limited number of patients in our study, the range of MaR is inherently smaller. Different infarct definition thresholds affect LGE MI size which influence the correlation between native T1 and LGE [30]. The use of a threshold algorithm for quantifying enhanced areas on native T1 maps would most likely also affect the correlation between native T1 and CE-SSFP. Also, the timing of CMR post-injury may affect the correlation, however earlier data have shown MaR to be stable over the first week in patients [9].

### Native T1-mapping and infarct size

An earlier study argued that native T1-mapping could be used to assess IS measured with LGE at two weeks after infarction [14]. That study was conducted in an experimental model with pigs undergoing 90 min of coronary occlusion, causing a transmural infarct with very little myocardial salvage and therefore very little difference between IS and MaR [14, 16]. On the contrary, our study demonstrates that native T1-mapping overestimates IS during the first week after reperfusion and rather, as earlier studies have shown, indicates that native T1-mapping depicts MaR [1, 27]. The viable area within MaR depends on time of ischemia and in our experimental model, with 40 min of occlusion leaves salvaged myocardium and therefore enables differentiation between IS and MaR. In an ischemic model with longer ischemia/time from ischemic insult the whole MaR would be necrotic/infarcted and elevated native T1 would then correlate with the infarct size [14]. The observed larger MaR-IS difference in patients than in the single-time-point animal group could be attributed to different infarct development between species. We choose our occlusion time in pigs, i.e. 40 min, to achieve approximately an infarct size equal to 50% of MaR [16]. As humans take longer time than pigs for 50% of the MaR to become infarcted, timely intervention in patients likely leads to more saved myocardium and thus a larger MaR-IS difference in patients. Methodological reasons for a larger MaR-IS difference in patients may be attributed to that native T1 maps were collected as a full-stack in pigs and only for three short-axis slices in patients. Patients were also awake during the CMR scan compared to pigs that were anesthetised, possibly minimizing slice position variability.

### Quantification of enhanced extent native T1-maps

This study used manual quantification of the enhanced extent on the T1-maps as there is a lack of validated algorithms. Threshold techniques such as Otsu or n standard deviations from remote have been used in previous studies for assessment of MaR and IS. Some of these quantitative techniques used for LGE images were recently shown to be highly unreliable as signal-to-noise ratio, number of standard deviations, and the area of the remote region of interest affects the resulting delineated area [34]. Also, validation studies are needed to establish reliable algorithms to delineate native T1 maps. Another factor with effect on relaxation properties is microvascular obstruction, seen in some of the study subjects. A previous study showed lower native T1 mapping values in microvascular obstruction or hemorrhage compared to the infarct boarder at 4–6 days post-MI [35]. However, the presence of MVO does not affect the delineation of enhanced MaR or LGE areas as MVO regions are located within enhanced regions.

### Comparison of T1-mapping sequences

In the animal part of the study, two different native T1-mapping sequences were analyzed i.e. MOLLI and SASHA. MOLLI is a commonly used sequence in the clinic but is dependent on sufficient breath-hold time, T2, off-resonance effects and magnetization transfer effects [36]. Also, MOLLI has been shown to have lower accuracy but higher precision and signal-to-noise ratio than SASHA [37]. Despite this, our study shows that both methods agree with CE-SSFP. This could be explained by that the difference in accuracy between MOLLI and SASHA did not play a role, because the enhanced extent was measured rather than the actual T1-values.

### Utility of native T1-mapping

The results from this study suggest that native T1-mapping MOLLI and SASHA may be used to obtain myocardial T1-values and MaR in clinical post-infarct patients. Today’s non-contrast protocols investigating edema in patients with contraindications for Gd rely on T2-weighted imaging [6]. However, T1-mapping is of interest for the clinician to differentiate between different diagnoses [38]. Therefore, this study supports the evidence that native T1-mapping can be used for gaining clinical diagnostic information, and simultaneously be used for assessment of MaR, causing a reduced scan time and enabling a wider patient inclusion with more comorbidities to represent the general population in future studies. Quantitative or semiquantitative T1-measurements may prove useful in future studies for differentiating between MaR and IS. This has been previously investigated using pre- and post-contrast measurements [13]. A fully non-contrast protocol only relying on quantitative native T1-mapping would be clinically useful to reduce scan time and enable imaging of patients with severe kidney insufficiency or pregnancy.

### Limitations

The results of this study should be viewed in the light of some limitations. The occlusion site consisted of LAD in all patients and animals. Hence, the conclusions in this study may predominantly be made regarding LAD infarctions. However, the validity of native T1-mapping against CE-SSFP in other culprit lesions assumably resembles the validity in LAD infarctions due to similar pathophysiology. In addition, only one slice per animal was collected in the serially imaged group. To counter for this, image full stacks were collected in the single-timepoint imaged group and were presented separately. Furthermore, the movement of the animal in and out of the scanner between CMR timepoints, makes the SAX slice location, to some degree, slightly different between timepoints. Therefore, conclusions of the edematous dynamic extent over time cannot be made from the native T1-mapping images in this study. However, the imaging of the same SAX slice location with native T1-mapping, CE-SSFP and LGE for each imaging timepoint enables comparison between the different methods. Thus, conclusions of interchangeability between the methods can be made at each CMR timepoint. The addition of T2-mapping as another comparator for edema could have increased the generalizability of the study. No histological reference standard for MaR and IS was available and CE-SSFP and LGE are used as imaging-based surrogates. Finally, this study is a single-center study with few patients mimicking experimental conditions, which may limit statistical robustness. Therefore, multicenter multivendor studies with larger sample sizes are needed.

## Conclusion

Our findings suggest that non-contrast native T1-mapping using MOLLI or SASHA agrees with CE-SSFP during the first week after an acute myocardial infarction when evaluating MaR in a pig model and in patients. Also, native T1-mapping is shown to overestimate the LGE hyperintense area, indicating the method to not primarily depict infarct size in the first week after an acute myocardial infarction.

## Supplementary Information

Supplementary Material 1.

## Data Availability

The datasets analyzed during the current study are available from the corresponding author on reasonable request.

## Abbreviations

CE-SSFP: Contrast-enhanced steady state free precession
CMR: Cardiovascular magnetic resonance
Gd: Gadolinium
IS: Infarct size
LAX: Long axis
LGE: Late gadolinium enhancement
MaR: Myocardium at risk
MOLLI: Modified Look-Locker Inversion recovery
MSI: Myocardial Salvage Index
SAX: Short axis
SAPPHIRE: Saturation Pulse Prepared Heart rate independent Inversion Recovery
SASHA: Saturation recovery Single-shot Acquisition
STEMI: ST-elevation myocardial infarction
T2W: T2-weighted
