# Supplementary material for: Native T1-mapping using cardiovascular magnetic resonance detects myocardium at risk during the first week following myocardial infarction in a swine model and in patients - comparison to contrast-enhanced cine steady-state free precession
Source: BMC Cardiovasc Disord. 2026 Jan 22;26:86. doi: 10.1186/s12872-026-05507-3 (PMC12849553; doi:10.1186/s12872-026-05507-3)
Supplement: Supplementary file 1 — Supplementary Material 1. [file 12872_2026_5507_MOESM1_ESM.docx]

**SUPPLIMENTARY MATERIAL**

| **Sedation** | **Medication** | **Dose** | **Company** |
| --- | --- | --- | --- |
|  | Ketamine | 30 mg/kg | Ketaminol, Intervet, Danderyd, Sweden |
|  | Midazolam | 5 mg/kg | Dormicum, Roche AB, Stockholm, Sweden |
|  | Atropin | 0.02 mg/kg | Atropin, Mylan, Stockholm, Sweden |
| **Anesthesia** | Isoflurane | Titrated to effect | Isoflurane, Baxter Medical AB, Kista, Sweden |

**Table S1.** Sedation and anesthesia medical protocol. Initiation of sedation was performed by intramuscular injection before intubation. Anesthesia was maintained by delivering Isoflurane through an inhalation anaesthetic conserving device (AnaConDa).

| **Husbandry** | |
| --- | --- |
| Farm conditions | The research animals were brought up together with other pigs on a farm until transportation to experimental facilities. |
| Living conditions | The research animals were always kept together in the research facilities and would never be held alone when awake. |
| Environmental enrichment | Hay was always accommodated to the animal boxes. |
| Sanitation | Boxes were cleaned daily, and hay was changed daily. |
| Food and water | Access to water was accommodated at all times. Food was accommodated daily on a regular basis. |

**Table S2.** Description of animal husbandry.

| **Humane endpoint** | **Action** |
| --- | --- |
| Signs of injury or disease. | Veterinarian contacted to decide further action. |
| Treatment resistant arrythmia during general anesthesia. | Animal sacrifice with pentobarbital or potassium chloride. |
| Reduced general condition between periods of general anesthesia | Veterinarian contacted to decide further action (continuation of experiment or sacrifice). |

**Table S3.** Description of humane endpoints.

| **Characteristics** | **Serially imaged group** | **Single timepoint imaged group** |
| --- | --- | --- |
| Number of animals | 7 | 4 |
| Spieces | Sus scorfa domesticus | Sus scorfa domesticus |
| Race | Landrace | Landrace |
| Age | 3 months | 3 months |
| Female | 4 | 4 |
| Weight | 39±3kg | 40±2 |
| Genetic modification status | No genetic modification | No genetic modification |
| Health status | Normal status at arrival | Normal status at arrival |
| Previous procedures | No previous procedures | No previous procedures |

**Table S4.** Characteristics of the pig study population.


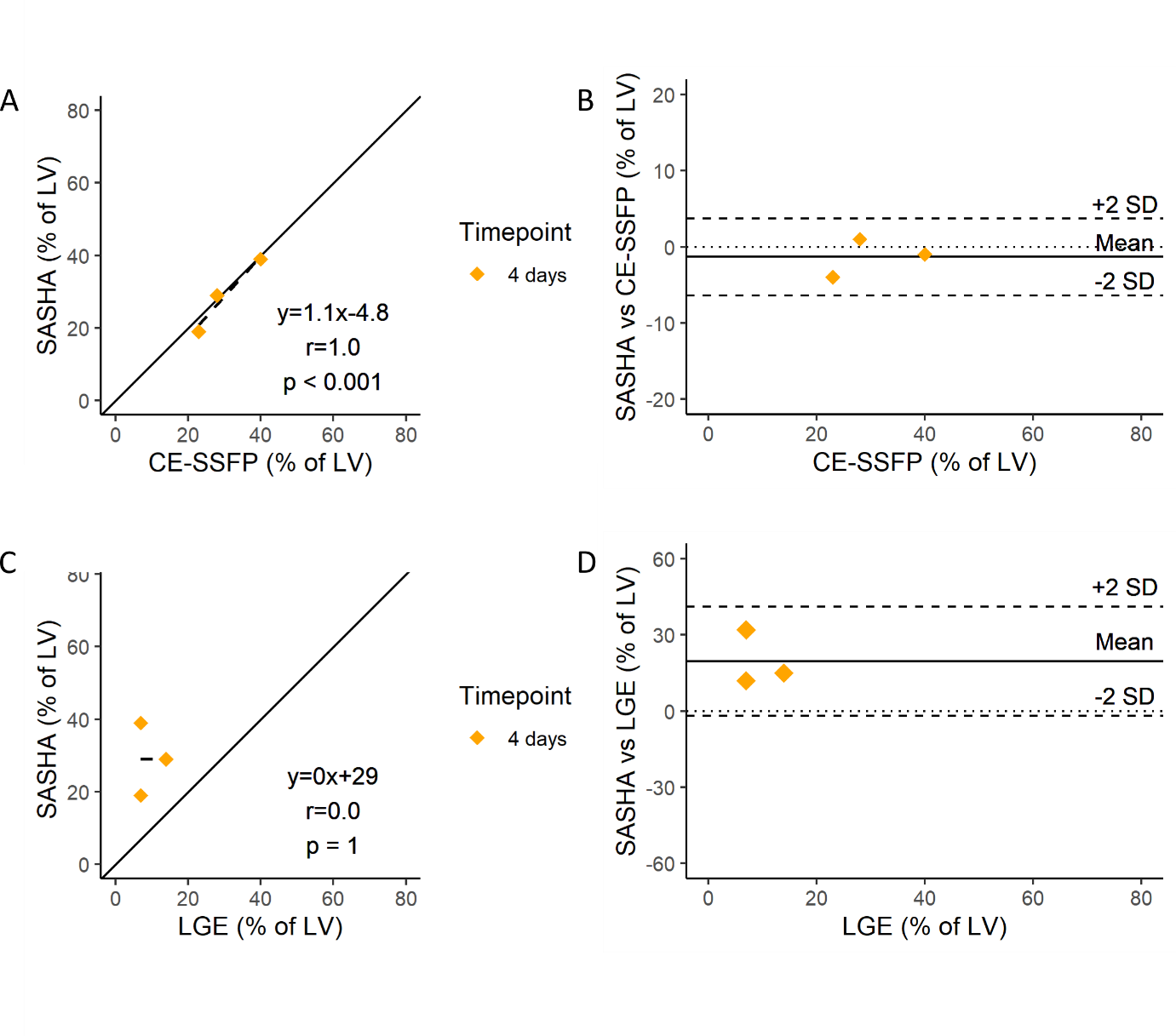
**Figure S1.** Native T1-mapping SASHA against CE-SSFP and LGE in the single-timepoint imaged group.
